# FiSH: fair spatial hot spots

**DOI:** 10.1007/s10618-022-00887-4

**Published:** 2022-11-17

**Authors:** Deepak P., Sowmya S. Sundaram

**Affiliations:** 1 Queen’s University Belfast, Belfast, UK; 2 L3S Research Center, Hannover, Germany

**Keywords:** Fairness in AI, Unsupervised learning, Hot spot detection

## Abstract

Pervasiveness of tracking devices and enhanced availability of spatially located data has deepened interest in using them for various policy interventions, through computational data analysis tasks such as spatial hot spot detection. In this paper, we consider, for the first time to our best knowledge, fairness in detecting spatial hot spots. We motivate the need for ensuring fairness through statistical parity over the collective population covered across chosen hot spots. We then characterize the task of identifying a diverse set of solutions in the noteworthiness-fairness trade-off spectrum, to empower the user to choose a trade-off justified by the policy domain. Being a novel task formulation, we also develop a suite of evaluation metrics for fair hot spots, motivated by the need to evaluate pertinent aspects of the task. We illustrate the computational infeasibility of identifying fair hot spots using naive and/or direct approaches and devise a method, codenamed *FiSH*, for efficiently identifying high-quality, fair and diverse sets of spatial hot spots. FiSH traverses the tree-structured search space using heuristics that guide it towards identifying noteworthy and fair sets of spatial hot spots. Through an extensive empirical analysis over a real-world dataset from the domain of human development, we illustrate that FiSH generates high-quality solutions at fast response times. Towards assessing the relevance of FiSH in real-world context, we also provide a detailed discussion of how it could fit within the current practice of hot spots policing, as read within the historical context of the evolution of the practice.

## Introduction

With sensing and tracking devices such as mobile phones and IoT becoming pervasive in this web-driven era, there is an abundance of spatial data across real-world settings. Within such spatial datasets, it is often of interest to identify geographically localized groups of entities that are of sufficient size and express a distinctive character so strongly that it is unlikely to have occurred by chance. To illustrate an example from our times, COVID-19 contact tracing apps accumulate large amounts of spatial data of people, of which some are known to have a COVID-19 infection. It would be of interest to automatically identify localized regions of high COVID-19 incidence, referred to as *hot spots* in contemporary reporting,^1^ so that the information could be channelized to health experts to identify causal reasons, or to public policy experts to develop a mitigation strategy for those regions.

**Fig. 1:**
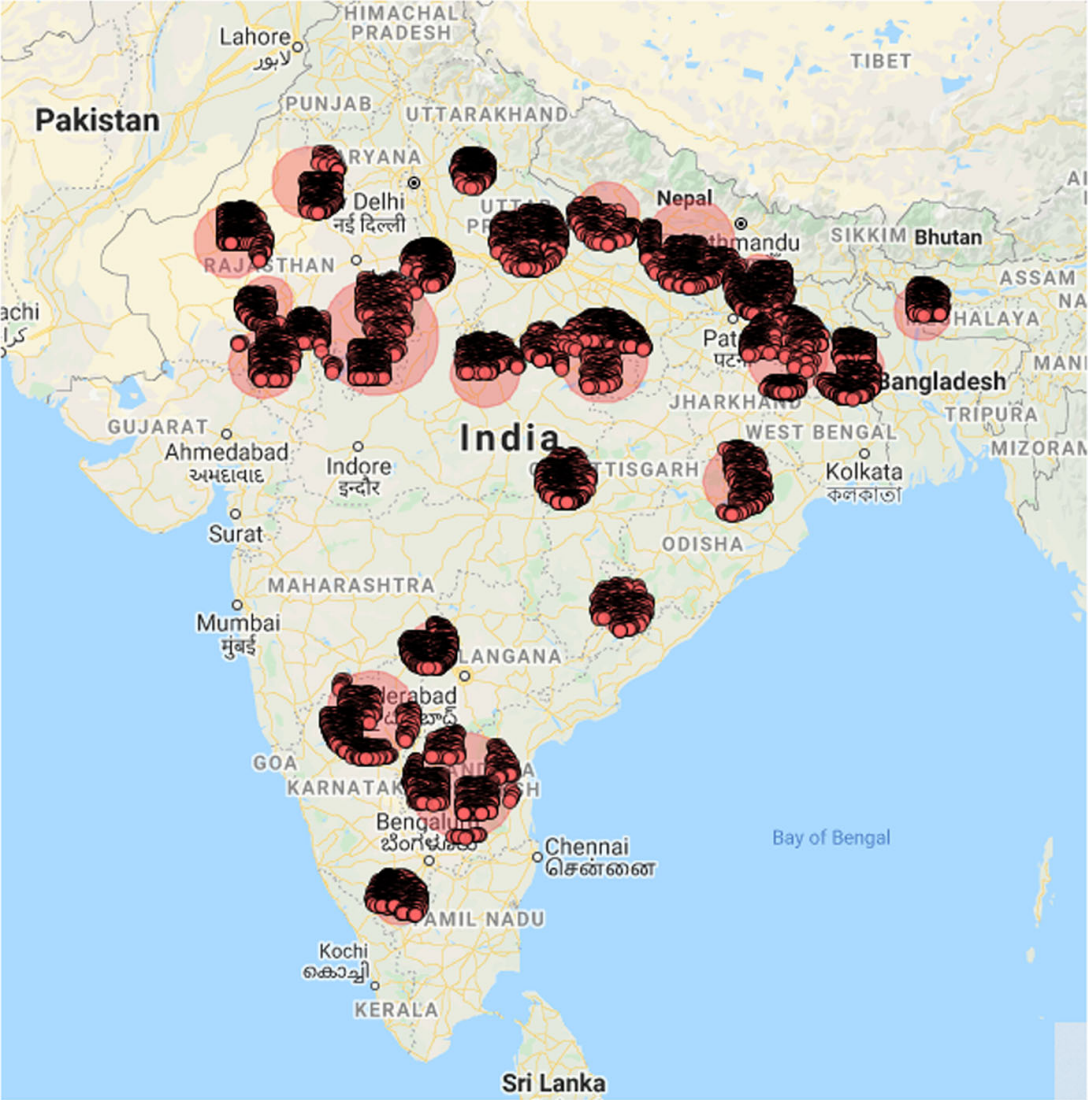
An illustration of hot spots of low educational achievement in India

While COVID-19 hot spots are characterized by *high disease incidence rates*, other web and new age data scenarios may call for different formulations of hot spot character, viz., *high crime rates* (law enforcement), *intense poverty* (development studies), *high mobile data usage* (mobile network optimization) and so on. For example, Fig. 1 illustrates hot spots of educational underachievement in India as identified from a human development dataset. In each case, identifying a set of hot spots would be of use so they could be subjected to an appropriate policy action. The unsupervised learning task of detecting spatial hot spots was pioneered by the spatial scan statistic (SSS) (Kulldorff 1997). The spatial scan statistic and its variants within the SaTScan^2^ toolkit have remained extremely popular for detecting spatial hot spots over the past two decades. While health and communicable diseases form the most popular application area of SSS (e.g., Pinchoff et al. 2015), they have been used within domains as diverse as archaeology (Wilczek et al. 2015) and urban planning (Helbich 2012).

### Fairness in hot spots

In scenarios where spatial hot spots are to be used to inform government and public sector action, especially in sensitive policy domains (e.g., law enforcement (Mohler et al. 2018), development), it is often important to ensure that the collective population subject to the policy action is diverse in terms of protected/sensitive attributes^3^ such as ethnicity, caste, religion, nationality or language, among others.

Consider hot spot detection on a crime database to inform policy action that could include sanctioning higher levels of police patrols for those regions. This would likely lead to higher levels of *stop and frisk* checks in the identified hot spots, and would translate to heightened inconvenience to the population in the region. Against this backdrop, consider a sensitive attribute such as ethnicity. If the distribution of those who land up in crime hot spots is skewed towards particular ethnicities, say minorities as often happens (Meehan and Ponder 2002), it directly entails that they are subject to much more inconvenience than others. These, and analogous scenarios in various other sectors, provide a normatively compelling case to ensure that the inconvenience load stemming from crime hot spot detection (and other downstream processing) be proportionally distributed across ethnicities. The same kind of reasoning holds for groups defined over other sensitive attributes such as religion and nationality. It is also notable that ethnically skewed hot spot detection and patrolling would exacerbate the bias in future data, leading to viscious cycles and runaway feedback loops (Ensign et al. 2018). Minor crimes are recorded in the data only when committed as well as observed. Thus, majority and minority areas with similar real crime prevalance, alongside minority-oriented patrolling, would lead to data that records higher crime prevalance in the latter. Second, even in cases where the intended policy action is positive (e.g., setting up job support centres for unemployment hot spots), the policy being perceived as aligned to particular ethnicities could risk social solidarity and open avenues for populist backlash (Greven 2016), which could ultimately jeopardize the policy action itself.

While considerations as above are most felt in policy domains such as policing and human development, these find expression in hot spot based prioritization in provisioning any common good. Ensuring fair distribution of the impact of any policy action, across sensitive attributes such as ethnicities, is aligned with the theory of *luck egalitarianism* (Knight 2009), one that suggests distributive shares (of inconvenience or benefits) be not influenced by arbitrary factors, especially those of ‘brute luck’ that manifest as membership in sensitive attribute groups such as ethnicity, religion and gender (since individuals do not choose those memberships are are often just *born into* one). Such notions have been interpreted as a need for orthogonality between groups in the output and groups defined on sensitive attributes, and has been embedded into machine learning algorithms through the formulation of *statistical parity* (e.g., Abraham et al. 2020).

In summary, there is an compelling case, as in the case of other machine learning tasks, for *hot spot detection to be tailored in a way that the population covered across the chosen hot spots be diverse along protected demographic groups* such as ethnicity, gender religion, caste and similar. We discuss some practical scenarios for fair hot spots further in Sect. 7.

### Our contributions

We now outline our contributions in this paper. *First*, we characterize the novel task of detection of fair spatial hot spots, for the first time. In particular, we outline a task formulation for enumerating a diverse sample of trade-off points in the noteworthiness-fairness spectrum, to suit diverse scenarios that require different trade-off points between noteworthiness and fairness. We note that straightforward solutions for the task would be computationally infeasible for even moderate dataset sizes. *Second*, we develop a method, FiSH, short for **F**a**i**r **S**patial **H**ot Spots, for efficiently enumerating sets of hot spots along the quality-fairness trade-off. FiSH works as a layer over any chosen fairness-agnostic spatial hot spot detection method, making it available across diverse scenarios and existing methodologies for those scenarios. *Third*, we outline a suite of evaluation measures to assess the quality and fairness of results for the novel fair spatial hot spots task. *Lastly*, we perform an extensive empirical evaluation over a real-world dataset over two separate contexts, which illustrates the effectiveness and efficiency of FiSH in identifying diverse and fair hot spots.

**Fig. 2:**
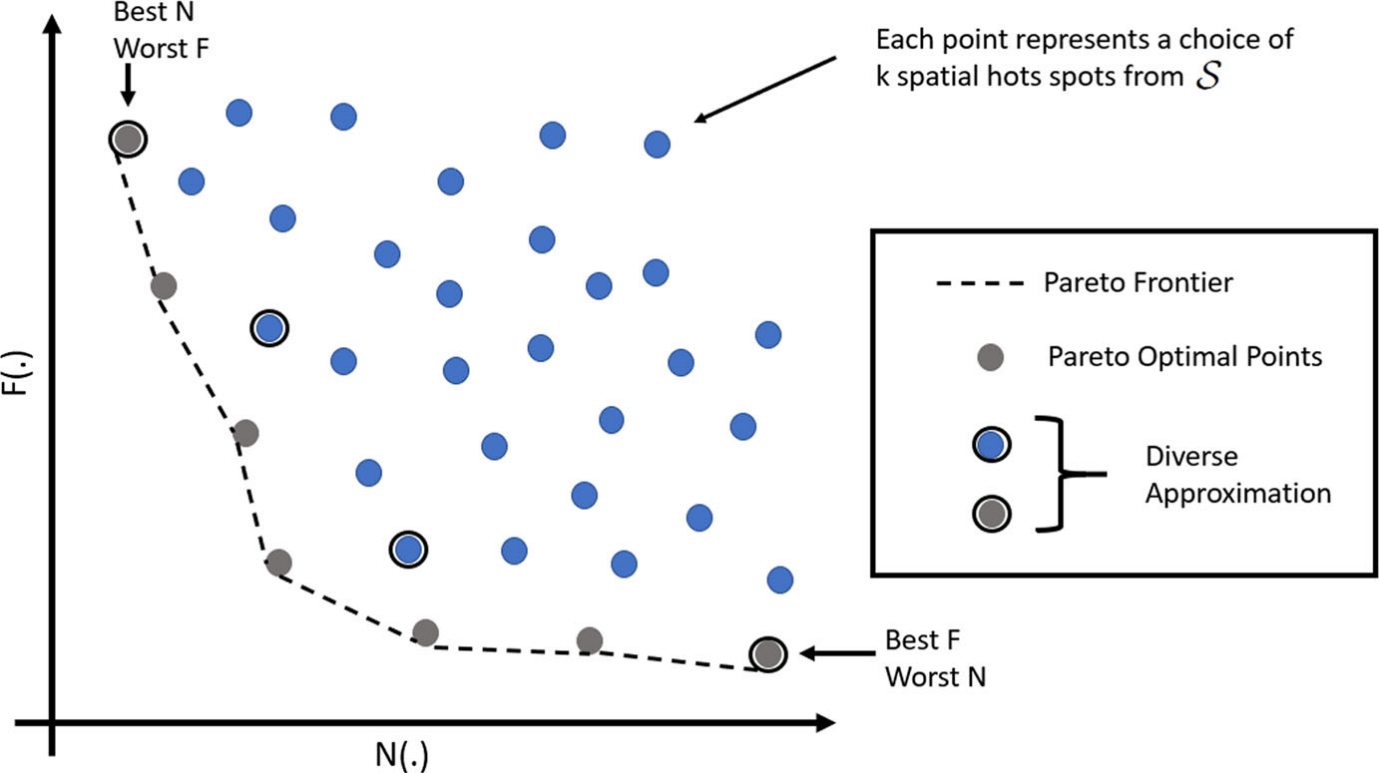
Illustration of the N–F space with *k*-sized subsets of ${\mathcal {S}}$. The Pareto frontier is marked with a dotted line. The circled points indicates a possible solution to the approximate $\tau$-dpe problem ($\tau = 4$). The exact $\tau$-dpe would comprise equally spaced points from the Pareto frontier

Given that fairness in spatial hot spots is a novel problem, we consider related work across two streams. We start by considering work on identifying *outliers* and *spatial hot spots*. These tasks are starkly different in terms of how the results are characterized. Outliers are determined based on neighborhood density over all relevant attributes in the data, whereas hot spots are determined based on *hotness* on a chosen hotness attribute (e.g., diseased, poor etc.) within an area of pre-specified type defined over spatial attributes. In particular, the notions of hotness attribute and spatial attributes are absent in the formulation for outlier detection, making them fundamentally different tasks. The interested reader may refer to Deepak (2016) (Fig. 2 and associated text) for a systematic analysis of the relationships between several allied tasks within this space. Despite being very different tasks, there are similarities in the overall spirit of outlier detection and hot spots, which makes outlier identification relevant to the interested reader. We start with a discussion on methods for the tasks of *outlier detection* and *spatial hot spots*, and then move on to work on fairness in machine learning as applied to tasks allied to ours.

## Related work

We now discuss related work along three streams viz., outlier detection, spatial hot spots, and fairness in unsupervised learning.

### Outlier identification

There have been a variety of problem settings that seek to identify objects that are distinct from either their surroundings or the broader dataset. The more popular formulations use the former notion, that of measuring contrast from the surroundings of the data object, i.e., making use of neighborhood density. LOF (Breunig et al. 2000) (and improvements (Kriegel et al. 2009)) consider identifying individual objects, aka *outliers*, which are interesting due to their (relatively sparser) spatial neighborhoods. It is noteworthy that these make object-level decisions informed purely by spatial attributes without reference to hotness attributes like diseased/non-diseased, as required for COVID-19 hot spot determination. SLOM (Chawla and Sun 2006) extends the object-level local neighborhood-based decision making framework to incorporate information from non-spatial attributes (e.g., age and gender of a COVID-19 patient), but do not consider hotness attributes such as diseased/non-diseased. Among outlier detection methods that assess the contrast of individual data objects with the dataset as a whole, the popular paradigm is to build a dataset level statistical model, followed by assessing the conformance of individual objects to the model; those that are less conformant would be regarded as outliers. Such statistical models could be a clustering (Yu et al. 2002), dirichlet mixture (Fan et al. 2011), or more recently, auto-encoders (Chen et al. 2017; Lai et al. 2020).

### Spatial hot spots

Spatial scan statistics (SSS), pioneered by Kulldorff (1997), are methods that identify *localized regions* that encompass multiple objects (in contrast to making decisions on individual objects, as in LOF) which *collectively* differ from overall dataset on chosen non-spatial hotness attributes (e.g. diseased, poor etc.). The focus is on characterizing regions which may be interpreted as *hot spots* due to the divergence of their character from the overall dataset. This makes SSS a markedly different task from outlier identification in specification, input data requirements, internal workings and output format. SSS spatial hot spots are vetted using a statistical likelihood ratio test to ascertain significant divergence from global character. This makes SSS as well as its various variants, as implemented within SaTScan, a statistically principled family of methods to detect spatial hot spots. While Kulldorff’s initial proposal is designed to identify circular hot spots, the framework has been generalized to identify arbitrary shapes in several ways; ULS (Patil and Taillie 2004) is a notable work along that direction. Other methods such as bump hunting (Friedman and Fisher 1999) and LHA (Telang et al. 2014) address related problems and leverage data mining methods. Despite an array of diverse research in identifying spatial hot spots, SSS methods have remained extremely popular. Just since 2020, there have been 2000+ papers^4^ that make use of SSS and other scan statistics within SaTScan. Our technique, FiSH, can work alongside any method that can provide an ordered output of hot spots, such as SaTScan methodologies.

### Fair unsupervised learning

While most attention within the flourishing field of fairness in machine learning (Chouldechova and Roth 2020) has focused on supervised learning tasks, there has been some recent interest in fairness for unsupervised learning tasks (Jose et al. 2020). Among the two streams of fairness explored in ML, viz., individual and group fairness (refer (Binns 2020) for a critical comparative analysis), most work on fair unsupervised learning has focused on group fairness. Group fairness targets to ensure that the outcomes of the analytics task (e.g., clusters, top-*k* results etc.) embody a fair distribution of groups defined on protected attributes such as gender, ethnicity, language, religion, nationality or others. As alluded to earlier, the most common instantiation of group fairness has been through the computational notion of *statistical parity*, initially introduced within the context of classification (Dwork et al. 2012). Group fair unsupervised learning work includes those on fair clustering (e.g., Chierichetti et al. 2017), retrieval (e.g., Zehlike et al. 2017) and representation learning (e.g., Olfat and Aswani 2019). While there has been no work on fair spatial hot spots yet, there has been some recent work on fairness in outlier detection which we discuss below.

#### Fair outliers

There has been some recent work on fair outlier detection. We start by outlining the core differences between outlier detection and hot spots to illustrate why fairness enhancements targeted at outlier detection would not be applicable for spatial hot spots. First, outlier detection often involves object-level decision making, whereas hot spotness can intrinsically be determined only at the level of object groups. Second, outlier detection methods do not make use of any non-spatial hotness attribute (e.g., diseased, poor etc.) to determine outlierness, whereas a *key non-spatial attribute is used to characterize hot spots*. The second difference makes algorithms for outlier detection contrast highly from those for identifying spatial hot spots. The first paper on fair outlier detection (Davidson and Ravi 2020) develops a framework for the task which is unique on multiple fronts. First, it is designed to be able to address unfairness over combinations of protected attributes leading to a deeper notions of fairness than methods that treat protected attributes one by one. Secondly, it leverages human know-how through seeking expert inputs on interpreting an explanation of potential unfairness. *FairLOF* (Deepak and Abraham 2020, 2021) focuses on automated group fair outlier detection, developing a technique that extends LOF (discussed above) for fairness. FairLOF adapts LOF to incorporate adjustments based on protected attribute memberships of the object in question and its neighbors, to ensure that protected groups are fairly represented among outliers. It may be noted that the protected attributes are used exclusively to embed fairness, and not to characterize outlierness. *FairOD* (Shekhar et al. 2020) makes a proposition of achieving group fairness (on protected attributes) while being expressly unaware of protected attributes at decision time (perhaps to avoid what is known as *formal* disparate treatment). A recent work (Zhang and Davidson 2021) on deep learning for fair anomalies/outliers proposes the usage of adversarial training and de-correlated representation learning to ensure that protected attributes are not correlated with outputs. To our best knowledge, there has been no prior work on fairness in detecting spatial hot spots or anomalous object groups of other kinds.

## Problem statement

Consider a finite dataset ${\mathcal {D}} = \{ \ldots , D, \ldots \}$. Each object *D* is associated with a set of spatial attributes such as (*x*, *y*) for a 2D space, or (*lat*, *long*) for locations of people. Further, each *D* is associated with a non-spatial *hotness* attribute $v \in \{0,1\}$ such as *diseased* (for epidemiology) or *poor* (for human development), which is used to determine spatial hot spots. *D* is also associated with protected attributes (e.g., ethnicity, religion) as we will see momentarily.

Consider a method for detecting spatial hot spots, such as spatial scan statistics, that is capable of providing a ranked list of top spatial hot spots, as ${\mathcal {S}} = [ S_1, S_2, \ldots , S_m]$. Each $S_i$ is associated with a spatial region $R_i$ (circular/spherical in the case of the basic SSS) such that the data objects (from ${\mathcal {D}}$) that fall within $R_i$ have a significantly different hotness profile than the overall dataset. For example, the population within $R_i$ may have a significant high (or low) incidence rate of poverty as compared to the whole population. Noteworthiness of spatial hot spots, analyzed statistically (as in SSS), is directly related to both the size of the population within the hot spot and the extent of divergence on the hotness attribute. ${\mathcal {S}}$ is the list of spatial hot spots ordered in decreasing statistical noteworthiness; thus $S_i$ is more noteworthy than $S_{i+1}$. When *k* (typically, $k<<m$) noteworthy spatial hot spots are to be chosen to action upon *without consideration to fairness*, the most noteworthy *k* hot spots, i.e., ${\mathcal {S}}_{topk} = [S_1, \ldots , S_k]$, would be a natural choice.

### Fair spatial hot spots

The task of fair spatial hot spots detection is to ensure that the *k* hot spots chosen for policy action, in addition to noteworthiness considerations as above, together encompass a diverse population when profiled along protected attributes such as ethnicity, religion, nationality etc, as motivated earlier. In other words, each demographic group is to be accorded a fair share within the collective population across the chosen hot spots. As mentioned earlier, this notion of *statistical parity* has been widely used as the natural measure of fairness in unsupervised learning (Chierichetti et al. 2017; Deepak and Abraham 2020; Bera et al. 2019). When the protected attributes are chosen as those that an individual has no opportunity to actively decide for herself (observe that this is the case with ethnicity, gender as well as religion and nationality to lesser extents), statistical parity aligns particularly well with the philosophy of *luck egalitarianism* (Knight 2013), as noted in Sect. 1.1.

We will use ${\mathcal {S}}_{fairk}$ to denote a set of *k* hot spots (from ${\mathcal {S}}$) that are selected in a fairness-conscious way. It is desired that ${\mathcal {S}}_{fairk}$ fares well on *both* the following measures:

1 $$\begin{aligned}{} & {} N({\mathcal {S}}_{fairk}) = \sum _{S \in {\mathcal {S}}_{fairk}} rank_{{\mathcal {S}}}(S) \end{aligned}$$

2 $$\begin{aligned}{} & {} F({\mathcal {S}}_{fairk}) = \sum _{P \in {\mathcal {P}}} Div_P({\mathcal {D}}, Pop({\mathcal {S}}_{fairk})) \end{aligned}$$

The first, *N*(.), relates with noteworthiness and is simply the sum of the ranks (ranks within ${\mathcal {S}}$) of the chosen spatial hot spots (*S* denotes a spatial hot spot, a set of items); $rank_{{\mathcal {S}}}(S)$ denotes the rank of *S* within the list ${\mathcal {S}}$. Lower values of *N*(.) are desirable, and ${\mathcal {S}}_{topk}$ scores best on *N*(.), due to comprising the top-*k* (so, $N({\mathcal {S}}_{topk}) = \sum _{i=1}^k i = \frac{k \times (k+1)}{2}$). The second, *F*(.), is a fairness measure, which requires that the population subset covered across the hot spots within ${\mathcal {S}}_{fairk}$ (denoted $Pop({\mathcal {S}}_{fairk})$) be minimally divergent to the overall population, when measured on a pre-specified set of protected attributes ${\mathcal {P}}$ (e.g., ethnicity, gender); $Div_P(.,.)$ measures divergence on attribute $P \in {\mathcal {P}}$. In other words, $Pop({\mathcal {S}}_{fairk})$ denotes the population subset chosen for policy action, and we are interested in measuring how divergent this population subset is, from the overall population, on the sensitive attributes. We use Wasserstein distance (Vallender 1974; Yoon et al. 2020) to compute divergence yielding the following formula:

3 $$\begin{aligned} Div_P({\mathcal {D}}, Pop({\mathcal {S}}_{fairk})) = Wass(Distrib_P({\mathcal {D}}), Distrib_P(Pop({\mathcal {S}}_{fairk}))) \end{aligned}$$

where $Distrib_P(.)$ is the normalized distribution of the population across the values of attribute *P*^5^. For example, if ${\mathcal {D}}$ has a 50:50 distribution on males and females and the sub-population in $Pop({\mathcal {S}}_{fairk})$ has a 55:45 distribution, $Div_P(.,.)$ for this case will be *Wass*([0.5, 0.5], [0.55, 0.45]), where *Wass*(., .) denotes the Wasserstein distance. When the distributions are identical, statistical parity is achieved, and $Div_P(.,.)$ evaluates to 0.0. The usage of Wasserstein measure as an evaluation measure for various fair ML algorithms (Wang and Davidson 2019; Deepak and Abraham 2020) and other contexts in fair ML (Miroshnikov et al. 2020) motivate its usage here. Intuitively, Wasserstein measures the minimal cost of transporting one distribution to another. $Div_P(.,.)$ can be easily modified to use another distance measure should such a motivation arise in a particular domain. As in the case for *N*(.), lower values of *F*(.) are desirable too. Since *F*(.) is an aggregate of $Div_P(.)$s, achievement of statistical parity over each of the protected attributes would naturally lead to *F*(.) evaluating to 0.0. Though lower, and not higher, values of *N*(.) and *F*(.) indicate deeper noteworthiness and fairness, we refer to these measures as noteworthiness and fairness to avoid introducing new terminology.

*Fair hot spots and fair ranking* The notion of fair hot spots is quite different, and arguably more complex, than tasks such as *fair ranking* that have been explored in literature (e.g., Zehlike et al. 2017). We briefly discuss the relationship between these tasks. Without loss of generality, fair ranking may be easily conceptualized within a hiring shortlisting scenario where the fairness need is to ensure a fair representation of gender and race groups within the pool of shortlisted candidates. Here each object—which is an applicant in the hiring scenario—has a membership within the protected group (e.g., male when gender is the chosen protected group), and group fairness is sought to be achieved within the shortlisted pool. Despite the superficial high-level structural similarity that fair hot spots seeks to re-order objects in ${\mathcal {S}}$ in accordance with fairness, the sharp departure is evident when one observes that what is sought to be ranked within the fair hot spots formulation are *hot spots*, which are not individual objects, but sets of objects. Hot spots relate to one another in set relationships (e.g., disjoint, overlapping, subset etc.), and each hot spot, due to comprising multiple objects, has a distribution of memberships over a protected attribute (e.g., gender). These two factors make the task of fair hot spots much more nuanced and intricate than fair ranking. It is also notable that given the structure of fair hot spots task, there is no direct dependency on the dataset size other than through the hot spots detection method employed.

### Diverse selection of ${\mathcal {S}}_{fairk}$ candidates

The noteworthiness and fairness considerations are expected to be in tension (an instance of the often discussed fairness-accuracy tension (Menon and Williamson 2018)), since *fairness is not expected to come for free* (as argued extensively in Kearns and Roth (2019)). One can envision a range of possibilities for ${\mathcal {S}}_{fairk}$, each of which choose a different point in the trade-off between *N*(.) and *F*(.). At one end is the ${\mathcal {S}}_{topk}$ (best *N*(.), likely worst *F*(.)), and the other end is a maximally fair configuration that may include extremely low-ranked hot spots from ${\mathcal {S}}$. These would form the Pareto frontier^6^ when all the $^{m}C_k$ (*k* sized) subsets of ${\mathcal {S}}$ are visualized as points in the 2D noteworthiness-fairness space, as illustrated in Fig. 2. Each point in the Pareto frontier (often called skyline (Borzsony et al. 2001)) is said to be *Pareto efficient* or *Pareto optimal* since there is no realizable point which is strictly better than it on ${\underline{\hbox {both}}}$ N and F measures. Thus, ${\mathcal {S}}_{fairk}$ candidates that are not part of the Pareto frontier can be safely excluded from consideration, since there would be a Pareto frontier candidate that is strictly better than it on both noteworthiness and fairness.

Each policy domain may choose a different point in the trade-off offered across candidates in the Pareto frontier, after due consideration of several available trade-off points. For example, policing may require a high-degree of fairness, whereas epidemiology interventions may be able to justify policy actions on less diverse populations based on the extent of supporting medical evidence. The Pareto frontier may be large (could contain hundreds of candidates, bounded above only by ${\mathcal {O}}(^{m}C_k)$) for a human user to fully peruse. The extreme case, where each of $^mC_k$ *k*-sized subsets of *C* are in the Pareto frontier, appears where no pairs are involved in a Pareto domination relationship. Thus, an obvious recourse would be to identify $\tau$ *diverse Pareto efficient candidates* (henceforth, $\tau$-dpe), where $\tau$ is a pre-specified parameter, so the human user may be able to choose appropriately from a varied set of possibilities. A natural and simple but incredibly inefficient solution would be to (i) enumerate the entire Pareto frontier, (ii) trace the sequence of Pareto efficient points from the top-left to the bottom-right (i.e., the dotted line), (iii) split the sequence into $\tau - 1$ equally sized segments, and (iv) take the $\tau$ segment end points as the result.

To summarize, the diverse candidate selection task outlined as $\tau$-dpe *requires a diverse set of Pareto efficient candidates in the N–F space, each candidate representing a* *k* *sized subset of* ${\mathcal {S}}$.

### Approximate $\tau$-dpe

It may be observed that it is infeasible to enumerate the $^mC_k$ subsets (e.g., $^{40}C_{10} = 8.5E$+8) in the N–F space just due to the profusion of possibilities, making exact $\tau$-dpe identification (as outlined in the four-step process in the previous section) infeasible for practical scenarios. This makes the task of identifying a close approximation of $\tau$-dpe results efficiently a natural alternative for a policy expert to examine the trade-off points and arrive at a reasonable choice of ${\mathcal {S}}_{fairk}$ to subject to policy action. This brings us to the *approximate* $\tau$-*dpe* task, which is that of efficiently identifying a close approximation of the exact $\tau$-dpe result. The set of circled points in Fig. 2 illustrates a possible solution to the approximate $\tau$-dpe task. All pertinent notations are outlined in Table 1 for easy reference.

It is useful to note the nature of likely usage contexts for $\tau$-dpe to provide some perspective on scalability. Whether it be the case of crime hot spots to inform police patrolling strategies, the case of poverty hot spots to inform public policy or even mobile data usage hot spots to inform network provisioning decisions, all of these are what we could call as *offline* tasks. In other words, while it is necessary to ensure that hot spots be identified in reasonable time, real-time responses are neither expected nor necessary. For example, while a response time in days or months (as would be required to traverse an exponential space) would be unacceptable, a response time of a few hours would be quite fine for practical scenarios. This is often common in unsupervised outlier detection as well; for example, the response time of a recently proposed random projection based outlier detection method (Bhattacharya et al. 2021) is of the order of several minutes or hours^7^. The approximate $\tau$-dpe formulation thus intends to bring the $\tau$-dpe task from the region of infeasible response times to the region of feasibility. Our method, *FiSH*, that addresses the approximate $\tau$-dpe task, is detailed below.

**Table 1:** Table of notations for easy reference

Notation	What it stands for
${\mathcal {S}}$	The ordered list of spatial hot spots used as
	Starting point for $\tau$-dpe task
${\mathcal {S}}_{topk}$	The subset of *k* most noteworthy hot spots from ${\mathcal {S}}$
${\mathcal {S}}_{fairk}$	*k*-sized subset of ${\mathcal {S}}$, a candidate for fair
	selection of hot spots
$N({\mathcal {S}}_{fairk})$	Sum of ranks of the spatial hot spots within
	${\mathcal {S}}_{fairk}$; lower denotes better noteworthiness
$F({\mathcal {S}}_{fairk})$	Deviation of ${\mathcal {S}}_{fairk}$’s population from dataset on
	Protected attributes; Lower denotes better fairness
*m*	Cardinality of ${\mathcal {S}}$
*k*	# hot spots from ${\mathcal {S}}$ Desired in each output candidate
$\tau$	Number of candidates desired in output
*b*	Beam width parameter used by *FiSH* (Sect. 4)

## FiSH: fair spatial hot spots

*FiSH* is an efficient heuristic-driven technique addressing the *approximate *$\tau$-*dpe* task outlined above. We first describe a systematic organization of the search space, followed by a heuristic method that traverses the space prioritizing the search using three considerations: *Pareto efficiency*, *diversity* and *efficient search*.

**Fig. 3:**
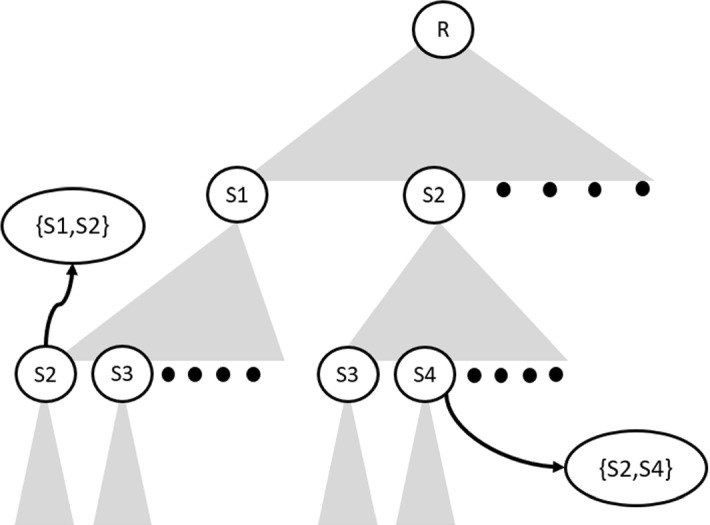
FiSH’s search tree: nodes at level *k* represent *k* sized subsets of ${\mathcal {S}}$, and form points in the N–F space (Fig. 2)

### Search space organization

Recall that we start with a noteworthiness-ordered list of spatial hot spots ${\mathcal {S}} = [S_1, \ldots , S_m]$. Our full search space comprises the $^mC_k$ distinct *k*-sized subsets of ${\mathcal {S}}$. We use the lexical ordering in ${\mathcal {S}}$ to organize these candidates as leaves of a tree structure, as shown in Fig. 3. Each node in the tree is labelled with an element from ${\mathcal {S}}$, and no node in the FiSH search tree has a child that is lexically prior to itself. Such a hierarchical organization is popular for string matching tasks, where they are called prefix trees (Yazdani and Min 2001). In devising *FiSH*, we draw inspiration from using prefix structures for skyline search over databases (Deshpande et al. 2009). Each internal node at level *l* (root level $=0$) represents a *l*-sized subset of ${\mathcal {S}}$ comprising the *l* nodes indicated in the path from root to itself. The lexical ordering ensures that each subset of ${\mathcal {S}}$ has a unique position in the tree, one arrived at by following branches corresponding to nodes in the subset according to the lexical ordering. The $^mC_k$ candidates would be the nodes at level *k*. It is infeasible to enumerate them fully, as observed earlier. Thus, *FiSH* adopts a heuristic search strategy to traverse the tree selectively to follow paths leading to a good solution (i.e., set of $\tau$ nodes at level *k*) for the approximate $\tau$-dpe task.

### FiSH search strategy

The exact $\tau$-dpe result set is characterized by *Pareto efficiency* and *diversity*, when applied over the $^mC_k$ candidates. The FiSH search strategy uses precisely these criteria as heuristics to traverse the search tree efficiently from the root downward. The core idea behind this search strategy is our conjecture that Pareto efficiency and diversity at a given level in the FiSH search tree would be predictive of Pareto efficiency and diversity at the next level. We operationalize this heuristic strategy using beam search, a classical memory-optimized search meta-heuristic (Steinbiss et al. 1994) that has received much recent attention (Wiseman and Rush 2016).

Having outlined all relevant notation and an overview of the search strategy, we now describe it in simple terms. FiSH starts its search from the root node, expanding to the first-level child nodes, each of which represent singleton-sets denoting the choice of a particular spatial hot spot from ${\mathcal {S}}$. This forms the candidate set at level 1 of the FiSH tree, ${\mathcal {C}}_1 = \{ \{S_1\}, \{S_2\}, \ldots \}$. These 1-sized subsets of ${\mathcal {S}}$ are then arranged in an N–F space as in Fig. 2. Note that the N–F space of 1-sized subsets is distinct and different from the N–F space of *k*-sized subsets (Fig. 2). The Pareto-efficient subset of ${\mathcal {C}}_1$ is then identified as $P({\mathcal {C}}_1)$. The candidates in $P({\mathcal {C}}_1)$ are then arranged in a linear sequence tracing the Pareto frontier from the top-left to the bottom-right point (similar to the illustration of Pareto frontier in Fig. 2). This linear sequence is split into $b-1$ equally spaced segments, and the *b* points at the segment end-points are chosen as $D_b(P({\mathcal {C}}_1))$, a *b*-sized subset of Pareto efficient points from ${\mathcal {C}}_1$. The candidate set at the next level of the tree search process, i.e., ${\mathcal {C}}_2$, is simply the set of all children of nodes in $D_b(P({\mathcal {C}}_1))$ (actually, the subsets of ${\mathcal {S}}$ that they stand for).

4 $$\begin{aligned} {\mathcal {C}}_2 = \bigcup _{c \in D_b(P({\mathcal {C}}_1))} children(c) \end{aligned}$$

It may be noted that ${\mathcal {C}}_2$ is a small subset of the set of all 2-sized subsets of ${\mathcal {S}}$, since only children of the *b* nodes selected from the previous level are selected for inclusion in ${\mathcal {C}}_2$. Next, ${\mathcal {C}}_2$ is subject to the same processing as ${\mathcal {C}}_1$ comprising:

1. identifying Pareto efficient candidates $P({\mathcal {C}}_2)$,
2. identifying a diverse *b* sized subset $D_b(P({\mathcal {C}}_2))$, and
3. following the children pointers,

to arrive at the candidate set for the next level. This process continues up until ${\mathcal {C}}_k$ whereby the Pareto frontier $P({\mathcal {C}}_k)$ is identified followed by the choice of $\tau$ diverse candidates which will eventually form FiSH’s result set for the approximate $\tau$-dpe task. This search strategy is illustrated formally in Algorithm 1. The one-to-one correspondence between nodes in the search tree and subsets of ${\mathcal {S}}$ allows us to use them interchangeably in the pseudocode.
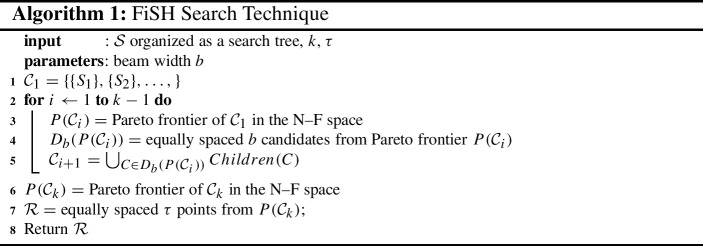


### Discussion

FiSH’s search strategy makes use of Pareto efficiency and diversity directly towards identifying a small set of nodes to visit at each level of the tree. Restricting the search to only *b* nodes at *each* level before moving to the next enables efficiency. Smaller values of *b* enable more efficient traversal, but at the cost of risking missing out on nodes that could lead to more worthwhile members of the eventual result set. In other words, a high value of *b* allows a closer approximation of the $\tau$-dpe result, but at a slower response time. It may be suggested that *b* be set to $\ge \tau$, since the algorithm can likely afford to visit more options than a human may be able to peruse eventually in the result set. The candidate set size at any point, and thus the memory requirement, is in ${\mathcal {O}}(bm)$. The computational complexity is in ${\mathcal {O}}(kb^2m^2)$, and is dominated by the Pareto frontier identification (which is in ${\mathcal {O}}(b^2m^2)$) at each level. While *b* is a controllable hyperparameter (likely in the range of 5-20), *m* can be constrained by limiting FiSH to work with the top-*m* result set (as ${\mathcal {S}}$) from the upstream spatial hot spot technique.

## Evaluating approx $\tau$-dpe results

Given that (approximate) $\tau$-dpe is a new task we proposed, we now describe novel evaluation metrics to assess the quality of *FiSH*’s results. Recall that, given the N–F space comprising all *k*-sized subsets of ${\mathcal {S}}$, the choice of $\tau$ equally spaced skyline candidates forms the result set for the exact $\tau$-dpe task that we propose in this paper. This result set, which we call *Exact*, is computationally infeasible for moderate datasets, but forms our natural baseline for measuring *FiSH*’s effectiveness. In other words, the results of *Exact* form the ground truth that *FiSH* seeks to approximate efficiently. Approximate $\tau$-dpe results from FiSH may be evaluated either *directly based on how well they approximate the expected results of the exact* $\tau$-*dpe task*, or based on *how well they adhere to the spirit of the* $\tau$-*dpe task* of identifying a diverse group of Pareto efficient subsets of ${\mathcal {S}}$. We now devise evaluation measures along the lines above. In what follows, we use ${\mathcal {P}}$ to denote the $^mC_k$ *k*-sized subsets of ${\mathcal {S}}$.

### Direct comparison

Let the result of the exact $\tau$-dpe task be ${\mathcal {E}} = [E_1, \ldots , E_{\tau }]$, and FiSH’s result be ${\mathcal {F}} = [F_1, \ldots , F_{\tau }]$. We would like the average distance between corresponding elements to be as low as possible.

5 $$\begin{aligned} DC({\mathcal {E}}, {\mathcal {F}}) = \frac{1}{\tau } \sum _{i=1}^{\tau } Eucl(E_i,F_i) \end{aligned}$$

where *Eucl*(., .) is the euclidean distance in the N–F space. Notice that when ${\mathcal {E}} = {\mathcal {F}}$, *DC*(., .) evaluates to 0.0. Given that *N*(.) and *F*(.) would be in different ranges, we will compute the distance after normalizing both of these to [0, 1] across the dataset. As may be obvious, smaller values, i.e., as close to 0.0 as possible, of *DC*(., .) are desirable.

### Quantifying Pareto-ness: coverage

A diverse and Pareto efficient set may be expected to collectively dominate most objects in the *N*-*F* space. Accordingly, we devise a measure, called *coverage*, that measures the fraction of candidates in ${\mathcal {P}}$ that are Pareto dominated by at least one candidate in ${\mathcal {F}}$:

6 $$\begin{aligned} Cov({\mathcal {F}}) = \frac{1}{\vert {\mathcal {P}} \vert } \sum _{P \in {\mathcal {P}}} {\mathbb {I}}(\exists F \in {\mathcal {F}} \vert F \succ P) \end{aligned}$$

where $F \succ P$ is true when *F* Pareto dominates *P*. A point Pareto dominates another if the latter is no better than the former on both attributes, excluding the case where both are identical in terms of their N–F co-ordinates. A candidate being dominated by another indicates that the latter characterizes an absolutely better trade-off point than the former (on both *N*(.) and *F*(.)). Thus, we would like the result set to be in a way that most, if not all, candidates are dominated by one or more candidates in the result set. *Cov*(.) is measured as a fraction of the candidates dominated, hence it is in the range [0, 1]. Full coverage (i.e., $Cov(.) = 1.0$) may not be attainable given that only $\tau$ candidates can be chosen in the result; instead, if we were to choose the entire skyline, we would get $Cov=1.0$ by design. Thus, the extent to which $Cov({\mathcal {F}})$ (FiSH’s coverage) approaches $Cov({\mathcal {E}})$ (coverage attained by the *exact* result) is a measure of FiSH’s quality. Coverage, being modelled using Pareto domination, may be seen as modelling *Pareto-ness* of FiSH’s result.

### Diversity of results

Given that our formulation of the approximate $\tau$-dpe task hinges on the idea that the candidates should be diverse (so that they may embody a variety of different trade-off points), diversity is a key aspect to measure the adherence of the solution to the spirit of the approximate $\tau$-dpe task. We model diversity as the minimum among pairwise distances between candidates in ${\mathcal {F}}$:

7 $$\begin{aligned} MD({\mathcal {F}}) = min \{ Eucl(F_i,F_j) \vert \{ F_i, F_j \} \subseteq {\mathcal {F}}, F_i \ne F_j \} \end{aligned}$$

Unlike the average of pairwise distances that allows nearby pairs to be compensated by the existence of far away ones, this is a stricter measure of diversity. On the other hand, this is quite brittle, in the sense just one pair of results being proximal would cause *MD*(.) to go down significantly; in such cases, the *MD*(.) would not be that representative of the overall diversity in ${\mathcal {F}}$. Hence, all the evaluation measures must be seen in cognisance of the others. Coming to desirable values of *MD*(.), we would like $MD({\mathcal {F}})$, which measures the lower bound of distances among elements in ${\mathcal {F}}$, to be as high as possible, and approach the diversity of ${\mathcal {E}}$, i.e., $MD({\mathcal {E}})$.

### Discussion

As obvious from the construction, lower values of *DC*, and higher values on both *Cov* and *MD* indicate the quality of FiSH’s approach. It is also to be seen that *Cov* and *MD* should be judged together, since it is easy to maximize coverage without being diverse and vice versa. *Cov* and *MD* requires all $^mC_k$ subsets of ${\mathcal {S}}$ to be enumerated, whereas *DC* requires additionally that the exact $\tau$-dpe results be computed. *This makes these evaluations feasible only in cases where such enumeration can be done*, i.e., reasonably low values of *m*. In addition to the above quality measures, a key performance metric that FiSH seeks to optimize for, is the *response time*.

## Experimental evaluation

We now describe our empirical study evaluating FiSH. In this section, we describe the dataset used, the experimental setup, our evaluation measures and our experimental results.

### Dataset and experimental setup

We describe the dataset and experimental setup in separate subsections.

#### Dataset

We used the Indian Human Development Survey (IHDS)^8^ dataset, a large-scale survey of India’s population conducted in 2011-12. This is one among very rare datasets—the only one we came across—that comprises personal information attributes along with locations. In particular, we used a random sample of 10000 individuals from the data with distinct locations. The location (lat, long) was determined through querying Google Maps based on the district and other location information available in the data. The binary *hotness* attribute was chosen as either (i) *(annual) income *< *It may be noted that100k*,^9^ or (ii) *education* < *2 years*. For each setting, we use *caste* and *religion* as sensitive attributes and *low* income/education as hot spot criterion. In other words, we would like to identify a set of spatial hot spots such that the population across them fare poorly on income (education) but religion and caste groups are fairly represented. These choices of attributes for hotness and fairness are abundantly informed by social realities in contemporary India; for example, caste discrimination remains rampant across India, including in urban settlements.^10^

#### Experimental setup

We used SaTScan Bernoulli model to discover hot spots. SaTScan is among the earliest and most popular hot spots detection methods; most improvements upon them are for more specialized scenarios. This backdrop makes usage of SaTScan most appropriate to showcase generality of *FiSH*, given that it operates as a layer over hot spots detection. We implemented *FiSH* as well as the *Exact* $\tau$-dpe computation (i.e., enumerate all $^mC_k$ subsets, find Pareto efficient frontier, and identify $\tau$ diverse subsets) on Python 3 on an Intel 64 bit i5-8265 at 1.6 GHz with 8 GB RAM. Unless otherwise mentioned, we use the following parameter settings: $m=20$, $k=5$ and $\tau = b = 5$. The source code for *FiSH* is available at https://github.com/Sowms/FiSH-Fair-Spatial-Hotspots.

### Overall comparison

We performed extensive empirical analyses over varying settings. We present representative results and analyses herein. Table 2 illustrates a representative sample of the overall trends in the comparison between *FiSH* and *Exact*. The low values of *DC* indicate that *FiSH*’s results are quite close to those of *Exact*, which is further illustrated by the trends on the *Cov* measure where *FiSH* follows *Exact* closely. For *MD*, we observe a 20% deterioration in the case of *Income*, and a 50% deterioration in the case of *Education*. We looked at the case of *Education* and found that the low value of *MD* for *FiSH* was due to one pair being quite similar (distance of 0.041), possibly a chance occurrence that coincided with this setting; the second least distance was more than three times higher, at 0.1349. On an average, the pairwise distances for *FiSH* was only 20% less than that for *Exact*. Across varying parameter settings, a 15-20% deterioration of *MD* was observed for *FiSH* vis-a-vis *Exact*. For the record, we note that the choice of first *k* hot spots from ${\mathcal {S}}$ as the result yielded $DC \approx 0.8$ and *Cov* 3 to 10 percentage points lower; this confirms that $\tau$-dpe task formulation is significantly different from *top-k* not just analytically, but empirically too.

**Table 2:** Comparative results (task setting: $\tau = 5$, $k=5$, $m=20$ and parameter setting: $b=5$ for FiSH); arrows denote whether low or high values are desirable

Setting	Method	DC $\downarrow$	Cov $\uparrow$	MD $\uparrow$	Time(s) $\downarrow$
*Income*	FiSH	0.112	0.995	0.034	23.11
	Exact	N/A	0.998	0.042	6536.54
*Education*	FiSH	0.045	0.987	0.041	23.87
	Exact	N/A	0.997	0.081	4413.78

**Table 3:** Scalability analysis: running time (in seconds) with varying *m*; *Exact* did not complete in reasonable time for $m>25$

	*Education*	
*m*	*FiSH*	*Exact*
15	17.83	840.37
20	23.87	4413.78
25	39.46	33151.91
30	49.28	
35	61.49	
40	71.09	

Apart from being able to approximate the *Exact* results well, *FiSH* is also seen to be able to generate results exceptionally faster (as expected, given the design of FiSH), a key point to note given that bringing the $\tau$-dpe task into the realm of computational feasibility was our main motivation in devising *FiSH*, as outlined in Sect. 3.3. In particular, *FiSH*’s sub-minute response times compare extremely favourably against those of *Exact* which is seen to take more than an hour; we will illustrate later that *Exact* scales poorly and rapidly becomes infeasible for usage within most practical real-life scenarios.

The *FiSH* versus *Exact* trends, reported in Table 2 is representative of results across variations in parameter settings. *FiSH* was consistently seen to record 0–10% deteriorations in *Cov*, around 15–25% deterioration in *MD*, and multiple orders of magnitude improvements in response time. The trends on the effectiveness measures as well as the response time underline the effectiveness of the design of the *FiSH* method.

### Scalability analysis

With *FiSH* being designed for efficient computation of a reasonable approximation of $\tau$-dpe results, it is critical to ensure that *FiSH* scales with larger *m*; recall that $m=\vert {\mathcal {S}}\vert$, the size of the initial list of hot spots chosen to work upon. Table 3 illustrates the *FiSH* and *Exact* response times with varying *m*. While *Exact* failed to complete in reasonable time (we set a timeout to 12 h) for $m>25$, *FiSH* was seen to scale well with *m*, producing results many orders of magnitude faster than *Exact*. In particular, it was seen to finish its computation in a few minutes even for $m \approx 100$, which is highly promising in terms of applicability for practical scenarios. Similar trends were obtained with scalability with higher values of *k* and $\tau$; *Exact* quickly becomes infeasible, whereas *FiSH*’s response time grows gradually.

**Fig. 4:**
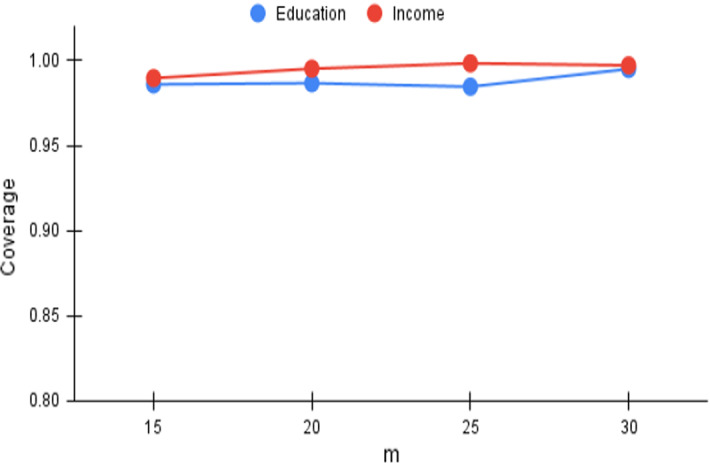
*Cov* versus m

**Fig. 5:**
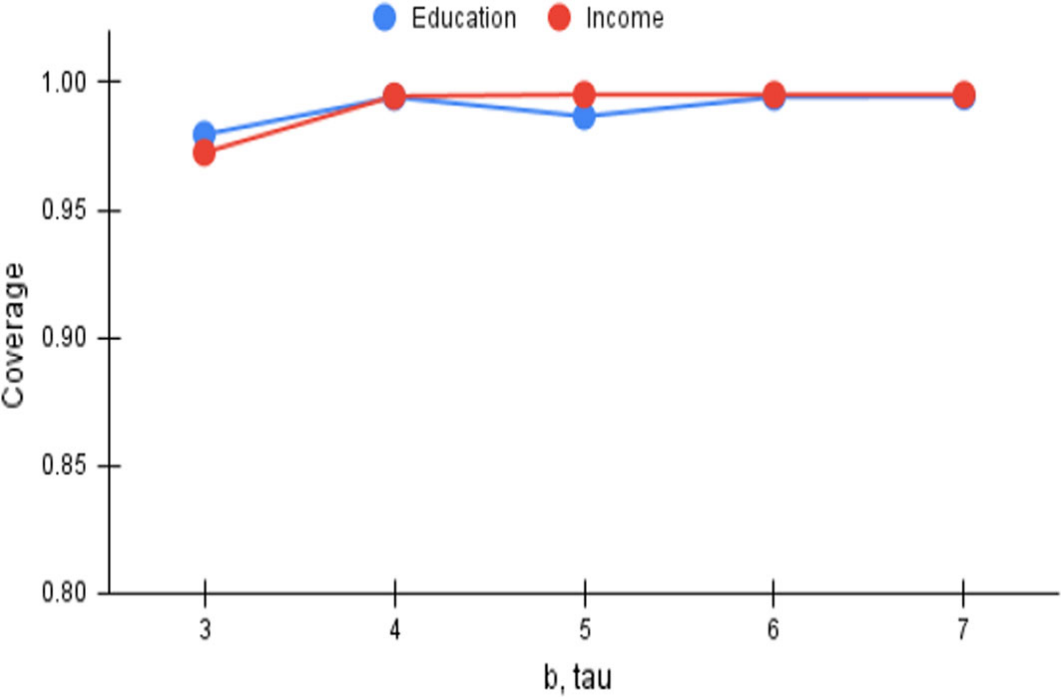
*Cov* versus b, tau

**Fig. 6:**
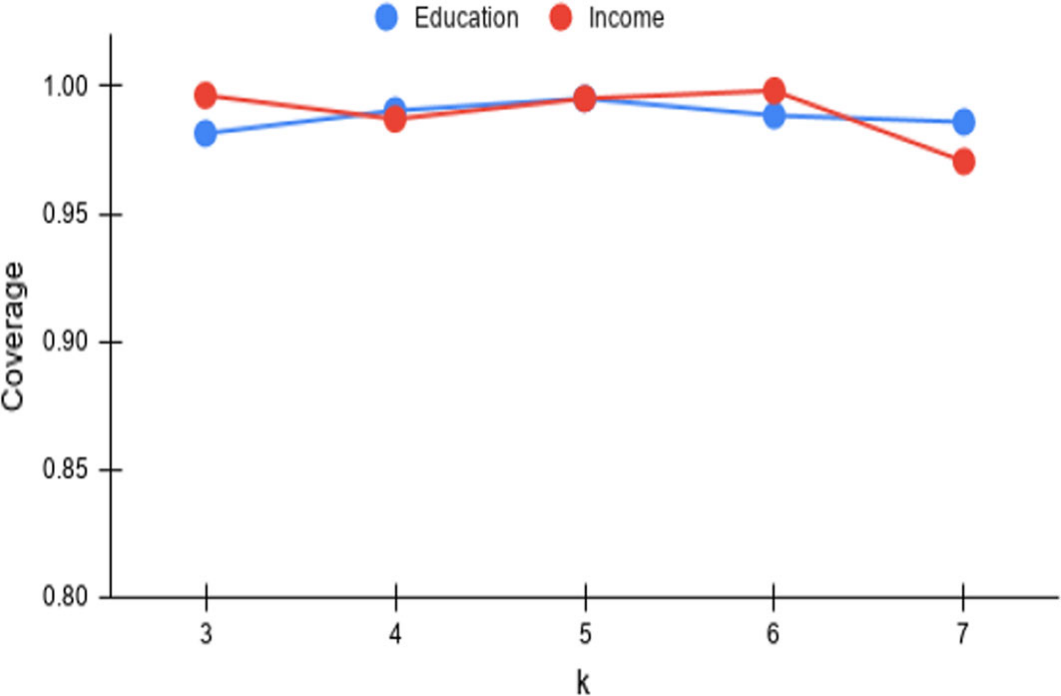
*Cov* versus k

**Fig. 7:**
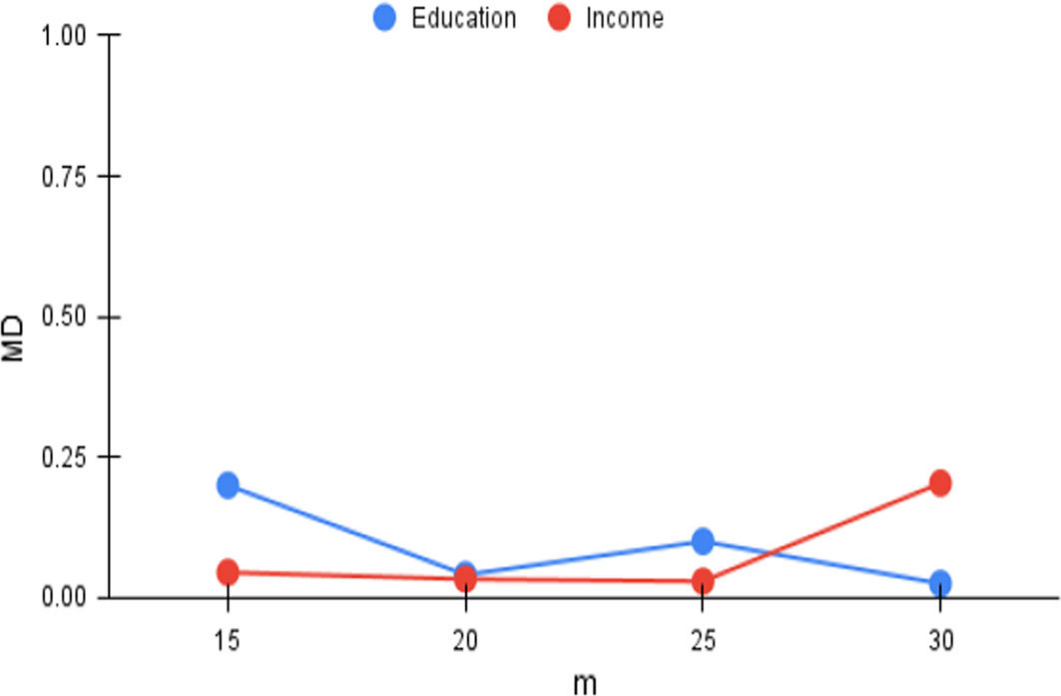
*MD* versus m

**Fig. 8:**
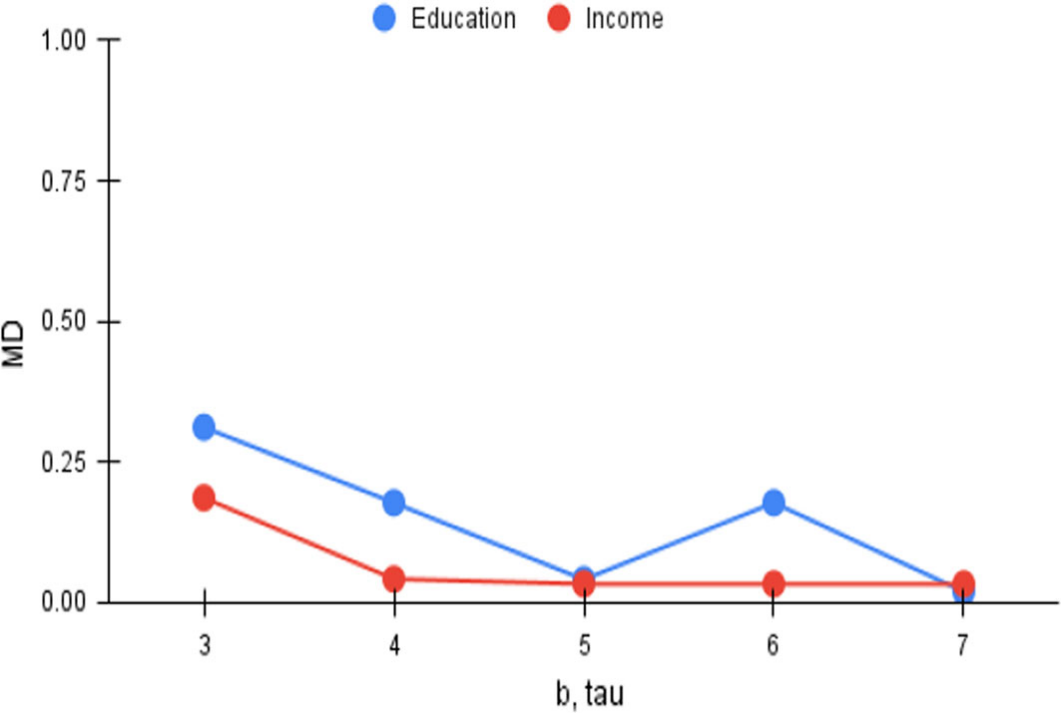
*MD* versus b, tau

**Fig. 9:**
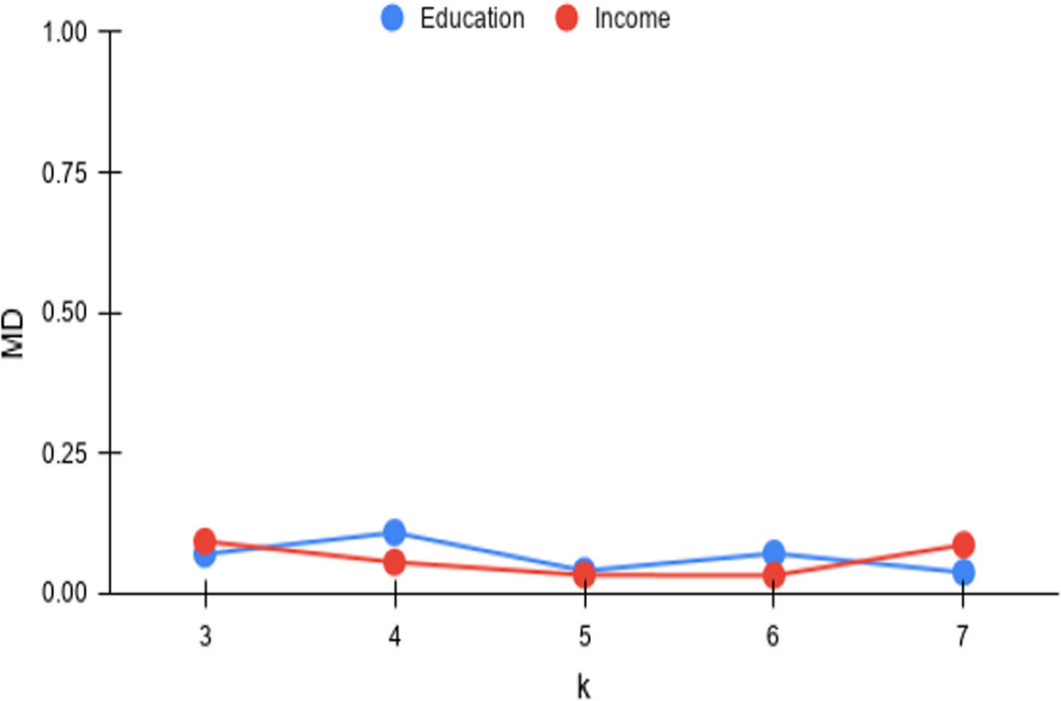
*MD* versus k

### FiSH fairness analysis

In addition to being able to generate good approximations of *Exact* at fast response times, it is also pertinent to analyze the fairness achieved over sensitive attributes by *FiSH* to get a sense of the levels of fairness achieved by *FiSH*. One may recollect that *FiSH* generates a set of results spread across the N–F spectrum. At the *F*airness end, we expect high degrees of fairness, where we expect that the distribution over sensitive attributes achieved over $Pop({\mathcal {S}}_{fairk})$ (Ref. Eq. 2) closely approximates that in the whole dataset; statistical parity is said to be achieved absolutely when these distributions are identical. The extent to which the F-end result’s distribution on the sensitive attributes reflects the dataset distribution is thus a key fairness metric for *FiSH*. Table 4 tabulates the distributions for this analysis. As may be seen therein, the distribution over the religion and caste for the *Income* case closely follows the full dataset distributions, with deviations under $2\%$ on an average. The corresponding deviations are just over $2\%$ for the *Education* dataset. These indicate that *FiSH* is able to provide a result option that achieves very high levels of fairness over the sensitive attributes.

### Analysis over varying settings

We now analyze the performance of *FiSH* in varying settings. This analysis helps us evaluate the sensitivity of *FiSH* to specific parameter values; for example, smooth movements along small variations in parameter values will help build confidence in the generalizability of *FiSH* across varying scenarios. With *Exact* being unable to complete running within reasonable amounts of time for higher search spaces (e.g., $m>25$, $k=7$, $\tau > 5$ etc.), we restrict our attention to *FiSH* trends over *Cov* and *MD*; this is so since results from *Exact* are necessary to compute the *DC* measure. Among *Cov* and *MD*, our expectation is that the brittleness of the *MD* measure, as noted in Sect. 5.3, could lead to more fluctuations in *MD* when compared to *Cov*, even when *FiSH* results change only gradually. We now study the trends with varying parameter settings, changing parameters one at a time, keeping all parameters at their reference settings from Sect. 6.1.2, except the one being varied.

#### Varying *m*

We now analyze the effectiveness of *FiSH* when operating over a larger set of SaTScan results, i.e., with larger values of *m* (recall $m=\vert {\mathcal {S}}\vert$). With the number of points in the N–F space being $^mC_k$, increases in *m* lead rapidly to much denser N–F spaces, and correspondingly larger search spaces. We vary *m* from 15 to 30 in steps of 5; the *Cov* and *MD* trends appear in Figs. 4 and 7 respectively. As expected, *Cov* consistently remains at high values, higher than 0.985, whereas there is higher volatility in the case of *MD*. The trends indicate that *FiSH* is not highly sensitive to *m* and the quality of its results varies gradually with varying values of *m*.

**Table 4:** *FiSH* fairness analysis

Scenario	Income		Education	
	Religion	Caste	Religion	Caste
Full dataset	$[0.270\ 0.730]$	$[0.210\ 0.790]$	$[0.310\ 0.690]$	$[0.200\ 0.800]$
F-end *FiSH*	$[0.267\ 0.733]$	$[0.217\ 0.783]$	$[0.303\ 0.697]$	$[0.195\ 0.805]$

#### Varying $\tau$

The number of trade-off points that is provided to the user, or $\tau$, is another important parameter in the $\tau$-dpe task. The beam size in *FiSH*, as observed earlier in Sect. 5.4, is intimately related to $\tau$, and may be expected to be set such that $b \ge \tau$. Higher values of *b* yield better results at the cost of slower responses; we consistently set $b=\tau$ in our result quality analysis. Higher values of $\tau$ enable choosing more points from the N–F space in the output, and this provides an opportunity to improve on *Cov*. However, choosing more points obviously would lead to deterioration in the *MD* measure that measures the minimum of pairwise distances. We vary $\tau$ (and thus *b*) from 3 to 7, and plot the *Cov* and *MD* trends in Figs. 5 and 8 respectively, which show gentle and consistent variations. As expected, *Cov* is seen to improve and saturate close to the upper bound of 1.0. *MD* on the other hand, is seen to deteriorate but stabilizes soon; the patterns are consistent except for the case of $\tau =5$ for *Education*, likely a chance occurrence as analyzed in Sect. 6.2.

#### Varying *k*

The third parameter of importance for the $\tau$-dpe task is *k*, which denotes the number of hot spots to be chosen within each trade-off point in the result. Increasing values of *k* (up to *m*/2) lead to larger number of points in the N–F space. With the number of trade-off points to be output pegged at $\tau$, achieving the same coverage would become harder with increasing *k*. This is in contrast with *MD* where there is no expectation of a consistent deterioration or improvement. From the *Cov* and *MD* plots in Figs. 6 and 9, the *Cov* is quite stable with a deterioration kicking in at $k=7$ (even there, *Cov* remains at $0.90+$), whereas *MD* remains consistent.

#### Setting *b*

The beam width, *b* in *FiSH*, offers a mechanism to trade-off effectiveness for efficiency. We experimented with varying values of *b* and found that the gains on effectiveness measures (i.e., *DC*, *Cov* and *MD*) taper off beyond $b > 2 \times \tau$. The response times were seen to increase with *b*; there are two ways in which *b* affects the complexity, one is by providing more candidates at each level (which increases linearly with *b*), and another by increasing the cost of Pareto frontier identification (which is in ${\mathcal {O}}(b^2)$). From the trends which indicated a linear trend between response time and *b*, it may be reasonably suspected that the former factor dominates.

**Fig. 10:**
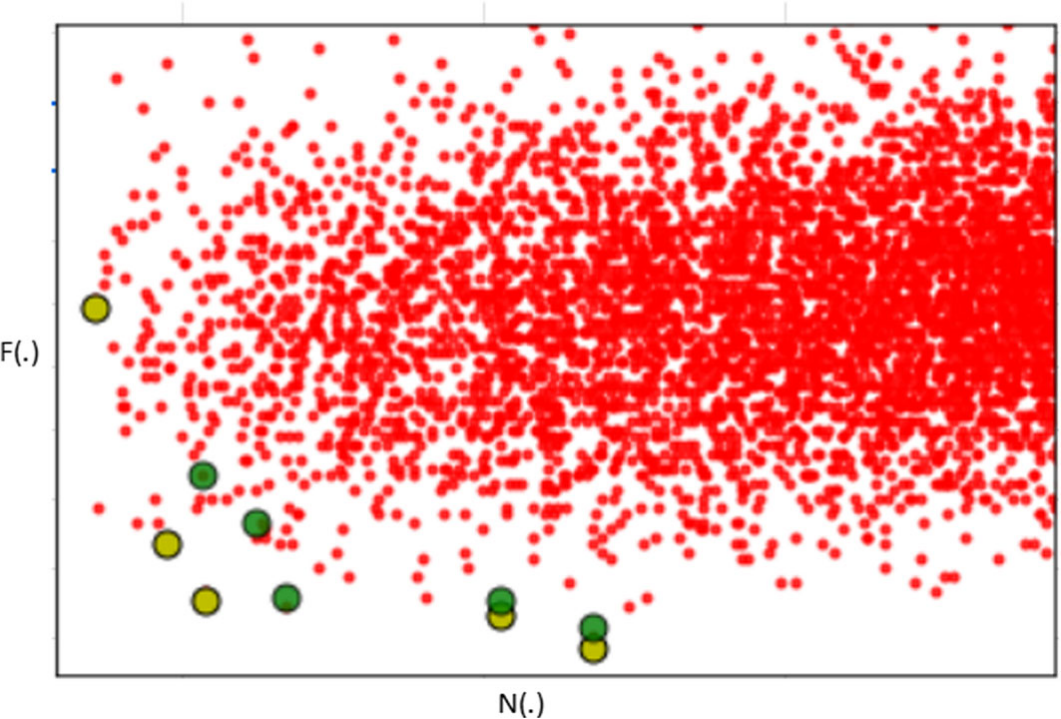
Example results; kindly view in color. *FiSH* results in green and *Exact* results in mustard yellow

### Example results in the N–F space

Having analyzed *FiSH* quantitatively, we now consider a qualitative evaluation of *FiSH* vis-a-vis *Exact*. Fig 10 illustrates the N–F space for our reference setting (Sect. 6.1.2) for *Income*, with results from *FiSH* (green points) juxtaposed against *Exact* results (mustard yellow) and other points in red. This result is representative of FiSH’s strengths and weaknesses. While three of five FiSH results are seen to be almost on the Pareto frontier, the others are only slightly inward. As in the case of any heuristic-driven method, FiSH may miss some good results; here, FiSH’s sampling misses out on the top-left region of the Pareto frontier, which explains the slight deterioration in *Cov* for *FiSH* when compared with *Exact*.

## Fair hot spots in practice

Against the backdrop of having discussed the technical details of our method, we now discuss the applicability of *FiSH* in practical scenarios. In particular, we motivate the role of fair hot spots—and methods such as *FiSH*—within the domain of crime prevention and surveillance, focusing on the specific context of hot spot policing.

### Hot spots policing: the context

Policing and crime prevention is a policy domain where hot spot detection is used in an apparent and visible manner, with much social and political implications. We scan the historical context of policing and crime prevention, and how the role of hot spot policing has evolved within it. Traditional notions of policing and crime prevention have focused on people, given the inarguable role of human agency in crime. With such people-focused policing, geographical considerations have traditionally focused on high-level and long-term decision making such as determining locations where police force needs to be stationed to ensure timely responses. The emergence of *place* as an important and explicit consideration within crime prevention arguably may be traced across four decades. A 1982 article titled *’broken windows’* (Wilson and Kelling 1982) foregrounded the idea that visible signs of anti-social behavior, such as broken windows, could be read as an evidence of enhanced crime-proneness within a place. This theory found much favor under the mayoralty of Rudy Giuliani^11^ in New York in the 1990s whose implementation of the theory included harsh crackdowns on minor crimes such as graffiti and turnstile jumping. This, probably among the first widespread place-focused policing in a wide scale, has led to much criticism and accusations of racism, with a 2007 book titled *’Why blacks fear America’s Mayor’* (Noel 2007) using the phrase *’one of the most racially divisive leaders in the nation’* to describe him. On the other hand, there has been much support for the approach, often citing sharp drops in crime in New York city under the Mayoralty of Giuliani (Langan and Durose 2003).

While *broken windows* is a place-based approach, modern approaches towards crime prevention often use the phrase *hot spot policing*^12^ explicitly, to denote a variety of place-based approaches in crime prevention. This relies on the assumption that police can be effective in addressing crime and disorder when they focus on small units of geography with high rates of crime. These hot spots and policing strategies and tactics focused on such areas are usually referred to as hot spots policing. The effectiveness of such strategies in deterring and/or reducing crime have been established through various studies (Sherman and Weisburd 1995). Over the past decades, the logic of hot spots policing has only gained in prominence, and has even been referred to as *’the* **law** *of crime concentration in places’* within an article (Braga et al. 2017) that says *’The empirical observation that a small number of micro places generate the bulk of urban crime problems has become a criminological axiom.’* The high impetus on hot spot policing is reflected in an increasing prioritization of government funding towards it^13^. The widespread emergence of the usage of AI-based tooling to assist place-based predictive policing (e.g., PredPol (Meliani 2018)) has played a significant role in the increased impetus on hot spot policing within contemporary society.

### Fairness and hot spots policing

As often observed in *fair machine learning* research, effectiveness and fairness are often in tension (Kearns and Roth 2019). The growth of place-based policing—often enacted through increased surveillance of hot spot locations within the city—has sharply coincided with concerns on systemic racism within policing. A recent book (Gordon 2022) uses the phrase *remaking of segregation* and—through a police shadowing study within a US context—draws a contrast between two sides of the city: *’one where rich, white neighborhoods are protected, and another where poor, black neighborhoods are punished’.* Such observations, one might recollect, dominated the public discourse during the *George Floyd protests of 2020*.^14^ While most studies have focused on US contexts, similar issues of bias in place-based policing have been brought up in other contexts within the Global South, such as the impact gradient of policing along caste lines within India (Narayan 2021). The developing narrative arguing against *new policing*, a term often used for technology-based and hot spot policing, and instead proposing a *newer policing*, one that is focused on rights, fairness and policing legitimacy, has seen growing acceptance. An article in *The Hill*^15^ says: *’The epidemic of police brutality—primarily affecting black males—can be linked to the history of a technique called hot spot policing, ...’*. The prominence of this narrative was acknowledged by a pioneer of hot spot policing, David Weisburd, whose 2016 article (Weisburd 2016) is titled: *Does Hot Spots Policing Inevitably Lead to Unfair and Abusive Police Practices, or Can We Maximize Both Fairness and Effectiveness in the New Proactive Policing?*. While Weisburd offers a positive view of hot spot policing in addressing this question through observing that hot spots policing encompasses a wide variety of implementation possibilities, he notes that *’Hot spots policing programs should be developed and implemented by police managers with the ideas of legitimacy and fairness in mind.’*

Apart from such extant observations that policing is being unfair and thus antithetical to a modern society, these additionally undermine the legitimacy of the police force, sowing the seeds for greater disharmony. We observe a sharp contrast between such emerging understanding within social science literature on the tensions between fairness and hot spot policing, and the lack of AI literature to provide enabling technological pathways towards bridging this tension. It is this deficit that our paper seeks to foreground and make initial strides towards addressing. Having outlined the context of hot spot policing and the need for fairness, we now consider how aspects of *FiSH* could be aligned with the challenges of fair hot spot policing.

### FiSH and fair hot spots policing

A key aspect of the fairness conceptualization within *FiSH* is that fairness is sought to be achieved *across the collection* of hot spots to be chosen for policy action rather than *within each* hot spot. This is based on the observation that imposing fairness conditions at the individual hot spot level could lead to an extremely constrained technical formulation. As emphasized in a recent interdisciplinary critical studies work (Webber and Burrows 2018), demographic identities in large urban locations are increasingly correlated with postcodes, making fairness imposition at the hot spot level impractical. This makes the fairness constraint best placed at the *across hot spots* level rather than any lower level of granularity. The next characterizing aspect of *FiSH* is the identification of multiple possible result sets comprising trade-offs in the noteworthiness-fairness spectrum. This is a conscious decision choice to distribute the agency in determining precise choice within the noteworthiness-fairness trade-off across the technique and the user, rather than being decided solely by the technology. These aspects, we believe, are highly aligned with the practical realities of policing, making *FiSH* a potentially important step towards fairness in hot spot policing.

### FiSH’s applicability gradient in hot spots policing

*FiSH* obviously has sub-contexts within hot spots policing where its applicability varies. While teasing out details of the applicability gradient requires on-field studies and correlation with pertinent literature (such studies are outside the scope of this work), we discuss some important considerations herewith. The most widely discussed contexts of policing fairness within the Western world relate to race, a protected attribute that happens to also be geo-correlated (Webber and Burrows 2018), making the across-hotspots fairness conceptualization within *FiSH* reasonably appropriate. We also note that there are similar cases within other geographies, such as fairness over the caste/religion dimension in India.^16^ However, in scenarios where fairness over other dimensions (e.g., affluence, education, and class) are more important and the geo-correlation on them may not be as pervasive within them, there may exist an opportunity to use a deeper notion of fairness viz., fairness constraints at the level of each hot spot. The next point where *FiSH*’s applicability gradient is apparent is to do with the nature and number of protected attribute to ensure fairness over. The current construction of *FiSH* addresses the categorical attribute case, but is amenable to be adapted to numeric attributes to accommodate protected attributes such as age and income, observed to be pertinent in the relationship between policing and gentrification.^17^ Towards using a numeric protected attribute, the entire numeric range can be bucketized, so it may be treated as a categorical attribute to leverage *FiSH* as it is. However, this may require bespoke bucketing mechanisms which are informed by domain knowledge. *FiSH*, in its present form, can only admit one protected attribute. *FiSH* may be extended—with non-trivial technical effort—to consider N–F trade-offs across a multi-dimensional space comprising one noteworthiness dimension and several fairness dimensions, one for each protected attribute.

## Conclusions and future work

In this paper, for the first time to our best knowledge, we considered the task of fair detection of spatial hot spots. In this web era where spatially-anchored digital data is collected extensively, spatial hot spot detection is used extensively to inform substantive policy interventions across a variety of domains, making fairness an important consideration within them. We characterized fairness using the popular notion of statistical parity when computed collectively over *k* chosen hot spots, and outlined the task of identifying a diverse set of solution candidates along the fairness-noteworthiness Pareto frontier. Observing the computational infeasibility of identifying exact solutions, we developed a method, *FiSH*, that performs a highly efficient heuristic-driven search to identify good quality approximate solutions for the task. We then formulated a suite of evaluation metrics for the novel task of fair hot spots. We perform an extensive empirical evaluation over a real-world dataset from the human development domain where fairness may be considered indispensable, and illustrated that FiSH delivers high-quality results, and offers good scalability, consistently returning results orders of magnitude faster than what is required to compute exact results. This illustrates the effectiveness of FiSH in achieving fairness in detection of spatial hot spots, and that it offers fast response times, making it appropriate for real-world scenarios. Through a detailed discussion on the context of hot spot policing, we illustrated how *FiSH* could provide significant strides in deepening fairness within place-based policing.

### Future work

While we have considered enhancing fairness by working upon a ranked list of spatial hot spots, FiSH extends easily to work over techniques that are capable of providing scores (in addition to ranks, which is basically an ordering over the scores) for each hot spot as well; we are considering evaluating FiSH’s effectiveness in working over such scored lists. Our formulation of diverse candidates assumes that the user may be interested equally in all parts of the noteworthiness-fairness trade-off space. However, in several cases, users may have a preference to exclude some parts of the space. For example, the maximum relaxation of noteworthiness may be bounded above in some scenarios. We are considering how user’s trade-off preferences can be factored into the FiSH search process to deliver diverse results within the sub-spectrum of interest.

## Data Availability

The dataset used is publicly available.

## Footnotes

1 https://www.nbcnews.com/news/us-news/map-track-summer-2020-coronavirus-hotspots-united-states-n1231332.

2 https://www.satscan.org/.

3 We use sensitive and protected interchangeably, in the context of attribute adjectives, throughout this paper.

4 https://scholar.google.com/scholar?as_ylo=2020 &q=satscan &hl=en &as_sdt=0,5.

5 While this design is motivated by categorical protected attributes which form the most popular type of protected attributes, this could be extended to other attributes as well.

6 https://en.wikipedia.org/wiki/Pareto_efficiency#Pareto_frontier.

7 They refer only to speedup rates in the paper; however, the KDD presentation has absolute response times.

8 https://ihds.umd.edu/data.

9 100k INR is approximately 1.35k$; India’s per capita income is $$\approx 2k \$$$.

10 https://www.economist.com/asia/2020/07/23/even-as-india-urbanises-caste-discrimination-remains-rife.

11 https://en.wikipedia.org/wiki/Rudy_Giuliani.

12 https://cebcp.org/evidence-based-policing/what-works-in-policing/research-evidence-review/hot-spots-policing/.

13 https://www.gov.uk/government/news/forces-given-funding-boost-to-increase-roll-out-of-hotspot-policing.

14 https://en.wikipedia.org/wiki/George_Floyd_protests.

15 https://thehill.com/blogs/congress-blog/civil-rights/265795-police-brutality-is-not-invisible/.

16 https://thewire.in/caste/police-casteist-communal.

17 https://housingmatters.urban.org/research-summary/neighborhoods-gentrify-police-presence-increases.

## Code availability

The dataset used is publicly available. Public. URL: https://github.com/Sowms/FiSH-Fair-Spatial-Hotspots.
